# The Causes of Failure in Dental Operations

**Published:** 1884-08

**Authors:** W. P. Horton


### The Causes of Failure in Dental Operations.

BY DR. W. P. HORTON.

Read before the Northern Ohio Dental Society.

Mr. President and Gentlemen of the Northern Ohio Dental Association:

The first subject provided for your consideration and digestion at this feast of reason is "The causes of failure in Dental Operations." In surgery an operation is defined to be "any methodical action of the hand, or of the hand with instruments, on the human body, with a view to heal a part diseased, fractured, or dislocated," and an operator is defined to be "the person who performs some act upon the human body by means of the hand or with instruments."

Your committee, however, has not formulated this subject on so broad a basis as to include general surgery, but qualified the word *operations* by placing the word *dental* immediately before it so that there might be no question as to the scope of the subject to be considered in this discussion at this time. Nor is it at all probable that your committee intended to include any consideration of artificial dentures and appliances, as you will see by reference to the programme that the third subject reads, "Prosthetic Dentistry, including artificial Crowns," so that by every fair rule of construction, the matter here presented for our consideration is the causes of failure of operations performed with a view to heal or restore any part diseased, fractured, dissolved away, or otherwise injured or lost, of the natural human dental organism. The teeth are not regarded as a part of the skeleton, being isolated from the bony structure, into which we find them inserted at their maturity by a thin membrane, known as the peridentium. In their anatomical structure, and in which they must be considered as separate bodies, they differ materially from all other organs; as, their shape, form, the adjustment of their several parts to each other, have reference solely to the varied functions they were designed to perform in the animal economy. Chemically considered, while they do not in their elements so widely differ from some other constituents of the body, they very materially differ in their proportions. And this for the reason that they are required to perform services that no other organ can, and the nature of the service requires different chemical proportions in their structure in order to fully prepare them to meet these demands, and, as an armor of defence against the attacks of enemies that are so by reason of their necessarily incongruous environments. I need not here enter further into particulars, as you are all familiar or ought to be familiar with their anatomy, chemical constituents and proportion, growth and location, beginning at an early period of fœtal existence, and proceeding generally in pairs, in regular order through deciduous and permanent, until the third molars are in position, and the entire denture completed at about the age of maturity of the body producing them; in whose service and for whose benefit they were designed to remain during the full period of the three score and ten years of earthly existence allotted to man.

Here I would be happy to leave them in all perfection of symmetry and glory, beautifying the human face divine, giving tone, sharpness, and precision to vocal utterances, performing the second process in feeding and sustaining the body; in short, filling the "ne plus ultra" of excellence. But, alas! our theme presupposes another, and not altogether so pleasant a condition of affairs among these organs as we would naturally suppose ought to exist at a time when the last and crowning act of creative wisdom was declared to be very good; for I believe that it is a well established fact that needs for dental operations have existed as long as the human family.

I said the teeth were regarded as no part of the human skeleton, yet they depend upon it for strength and support, and when removed from it, cease to exist as sensative vital organs, and become powerless to perform any of the various functions they were designed to, so that to perfectly fulfill the purposes of their creation, they must be kept in the places of their nativity, with all their surroundings complete and in perfect accord. And here before proceeding to consider any of the reasons for failures in dental operations, it will be proper to describe some of their more important connections with the general system, that we may better appreciate the difficulties to be overcome. They are connected with the skeleton by an articulation called "gomphosis," and I believe the only articulation of the kind found in the human body, all others being more or less movable. They are inserted into the superior and inferior maxilla in this manner, about three-fourths of their entire length; their covering to that extent, consists of jaw proper, alveolar processes, and the external tissue, including the mucous membrane. This last dips down into the socket forming the perio-ostio, perio-cement membrane surrounding the root and lining the socket, and furnishing nerve tissue and nutrition to the tooth externally. They are connected with the circulatory and nutritive system, by means of the apical foramen through which extensions of the maxillary arteries, superior and inferior, and out through which the veins extend to convey the return current for purification. And last, though perhaps not least, they are connected with all other organs through this same foramen by the extension of the great nervous system. Upon these organs, at the first glance, apparently so isolated, so lonely and desolate, with their environments and entangling alliances, physiologically and pathologically considered, has been and is to continue to be built the whole system of theory and practice of Dental Surgery.

It would seem that in presenting matters for our consideration, your committee has purposely avoided confining their subjects to the usual narrow limits of specifics, such as the treatment of alveolar abscess, periodontitis, gold as a filling and the like, and presented these subjects for discussion in more general terms. This is well; for while there are here and there workers in our vineyard, who, in their upward growth, have passed above the level of mere manipulative drudgery, and are putting forth all their energies, and using all and every means in their power in bringing out and formulating these specific features, for the good of the profession, and through that the well being of humanity, very few, in our society, have passed up into this higher atmosphere. If they have, they have not heretofore let their light shine so that it has come to pass that when these specific subjects have been presented, but little has been said in the way of discussion, for the reason that any remarks in a general way might be considered out of order. But here "No pent-up Utica contracts our powers, for the whole boundless field of Dental operations is ours," for consideration and thought in presenting some reasons for failures in Dental operations. I do not wish to be understood as making any attempt at exhausting the subject, or in any way disparaging or belittling other reasons that may occur to gentlemen, and which I hope and have no doubt they will offer when their time comes. Nor shall I persue any strictly methodical line; but if I succeed in provoking criticism and calling forth lively discussion so that both old and young may give us the benefit of their varied experience, I shall have accomplished the purpose for which I penned these lines. The first reason I shall offer is want of a proper diagnosis. You have a patient in the chair, to have temporary teeth filled or extracted, you make careful examination of the mouth, learn what specifically is to be done, and with the consent of the patient, parent, or guardian, proceed to perform what your diagnosis dictates. If the diagnosis be right, the operation will be a success; if wrong, it may be a failure.

You have a patient in the transition state, with both temporary and permanent teeth to be dealt with. In such cases your examination must take a wider range, including age, sex, temperament, and habits, and sometimes in addition, the wildest, crudest theories imaginable on the part of parents or guardians as to the present and future of the teeth, so that you not only are obliged to determine the case for the patient, but very often to enlighten and silence or overcome in the minds of those having control of the patient the most unreasonable prejudices, before you are permitted to perform what your diagnosis dictates ought to be done. If it be extracting, what teeth to extract, and what will be the effect upon the permanent teeth, contour of the face, and articulation, if this or that one is removed. If it be filling, what material is best, and how the operation can be best performed. If the diagnosis is right, all may be well. If wrong, an irreparable injury may be inflicted. We pass on to an older person, one beyond the transition state, whose teeth are in situ. In this case you have teeth to be filled. With what material, and how? If it be simply filling teeth in the mouth of an apparently robust, strong healthy constitutioned patient of either sex, between the ages of twenty and thirty five, and you have *carte-blanche* as to the kind of material and the price you shall charge, you cannot get far from the right result to the patient and credit to yourself, if you have the intelligence to know what a good filling is, and understand the necessary elements of material, skill, and labor that enter into, and the will and the physical ability to perform a good operation. But just here may step in a question of finance or prejudice which you will be obliged to settle to the satisfaction of the patient, if not, to yourself—and which may or may not materially detract from the operation as the highest standard of excellence dictated by your own judgment. But the next patient comes with what in common parlance is denominated toothache, for every pain in or around the teeth, from whatever cause, is denominated toothache. Now your range must be broader, deeper, and more searching—from what emanates the trouble complained of; is it from a tooth or teeth, or reflex from some other diseased organ, intimately connected with them? If from a tooth or teeth, can the offending member be saved and made useful? If from slight caries with sensitive dentine, yes. If from exposed pulp, what is its local and what its systemic condition? Is it a recent exposure; or has it proceeded to some other state—from simple irritation to suppuration? And what and how far are other organs and tissues involved and affected? And what about the circulation and the nutrition? If defective, from what cause and how about any peculiar diathesis? These and more than these questions having been settled and the treatment fully determined, your best endeavor may be defeated through the determinate will of the patient in a contrary direction. Either through a lack of information, neglect, want of a proper estimate of the value of the offending member, or want of confidence in the ability or integrity of the operator.

And now the field is before you, from the tiny puling suckling to the octogenarian. Some with good constitutions, strong and vigorous, with all their organs except dental performing their functions properly. Others in all stages of defective nutrition, debilitation, and weakness. From the rustic to have his tooth "*drawd*" to the neglectful one whose mouth requires a thorough preparation for the insertion of a full artificial denture. From the careful one to have the tiny speck of tartar removed and the minutest cavity filled at once, in the best manner, to the less careful and considerate, who, as a last resort, requires large and complicated operations to prolong the existence of the natural organs for yet a little while. All, all rush upon you, pell-mell, seeking advice or assistance at your hands. How shall the advice be given and how shall the assistance be rendered to the best good of the patient; for it is your duty (and mine) to declare the whole council, whether men will hear, or forbear. This requires a thorough preliminary preparation before entering upon the practice of dentistry, and should be as thorough as is required to practice general medicine or surgery, or any specialty of surgery.

There is now no excuse for ignorance. The means of education are abundant, to be had at reasonable outlays of time and money. So with head, hand, and heart, enlightened, any young man may enter upon the practice of dentistry fully persuaded that in the great lexicon of life there is no such word as fail. And at the close of a long and tedious career in efforts to do good and get good, should it turn out that, while from circumstances beyond his control, he had not in all cases reached that high point of excellence so desirable and at which he had aimed, yet if any amount of good had been bestowed upon suffering humanity it could hardly be charged as a failure.

